# What semantic analysis can tell us about long term trends in the global STI policy agenda

**DOI:** 10.1007/s10961-022-09959-5

**Published:** 2022-08-04

**Authors:** Leonid Gokhberg, Dirk Meissner, Ilya Kuzminov

**Affiliations:** National Research University Higher School of Economics, Myasnitskaya Ulitsa, 20, Moscow, Russian Federation 101000

**Keywords:** Science policy, Technology and innovation policies, Evidence based policy, OECD Working Party on Technology and Innovation Policy, Big data analysis, Semantic analysis, Text mining, International policy agenda, L5, F00, O1, O3, O4

## Abstract

The scope, complexity and the “volume” of knowledge accumulated render producing an overview of the core themes of science, technology and innovation policies difficult. Reviews of this policy domain mostly either refer to general issues without deep immersion into details or focus on specific narrower aspects. The paper uses semantic analysis to identify major themes of science, technology and innovation policies over the last three decades and to trace their evolution towards current policy setting. We use semantic tools for processing and analysing documents produced by one of the major and highly reputable international expert bodies, the OECD Working Party on Technology and Innovation Policy. We show that selected themes remain in the mainstream even though being affected by regular policy adjustments and refinements and which disappear or appear with new challenges and expected solutions. Other themes appear niche or exotic themes which are under discussion for some time only.

## Introduction

Science, technology and innovation (STI) policies aim at contributing to relevant conditions at the international, national and regional levels to ensure long term economic growth and social welfare. They have a horizontal character touching upon diverse policy fields, related to health, education, employment, migration, trade, taxation, infrastructure, investment, SMEs, competition, etc. Thousands of policy documents and analytical reports issued over decades across the globe reflect those considerations. Consequently, most reviews of this policy domain either refer to general issues without deep immersion into details or focus on specific narrower aspects. Thus, in order to understand the evolution of STI policies over time and identify major themes attracting stakeholders’ attention, there is a need for high-level, synthetic overviews that would also connect to the more granular levels.

Various arenas and institutions around the world bring STI policy makers and academics together to discuss national policy approaches and ensure their international co-ordination, find inspiration for national policies and learn from other countries’ experiences. One of the most influential and internationally renowned bodies is the OECD Working Party on Technology and Innovation Policy (WP TIP), which has met more than 50 times since 1993, gathering twice-yearly all OECD member countries and partner economies which are represented by renowned experts delegated by relevant national and international agencies and academia.^1^ The results of these meetings are documented in project reports, working documents and publications, which mirror the opinions of all Working Party members across countries because they are typically approved by consensus. The themes targeted by WP TIP over the years are fairly reflective of the interests of policy makers and orientations of the global policy discussion. Although not necessarily covering immediately the entire field of STI policies, they therefore give an open window on the field and provide the material needed for a relevant meta review.

In this paper, we apply semantic analysis to proceedings of the WP TIP meetings^2^ to investigate the evolution of its working themes over time—which ones remained in the mainstream even though affected by policy adjustments and refinements, and which ones disappeared or newly emerged, sometimes under different umbrellas. State-of-the-art text mining methodologies rest on computational linguistics, machine learning and network analysis. The respective algorithms and tools used are further described below. Aside from drawing new perspectives on STI policies by applying these innovative approaches, we show how semantic tools provide new opportunities for policy analysis.

The analysis of WP TIP documents is particularly relevant given the long history and high standing of this group, and its respective outputs which provide a solid information basis about relevant STI policy related themes at a given time. The work was undertaken at the occasion of its 50th meeting, the TIP asked several expert groups to implement semantic analysis techniques (also known as “text mining” or “natural language processing”, NLP) on its corpus, with the view to draw a synthetic view of its achievements and evolution over the years.

We find that two separate but complementary policy frameworks have played an important role in the WP TIP’s international STI policy debates:

- a structural, systemic one, emphasizing the linkages between actors, and
- an economic, financial (both budgetary and private) one focusing on the allocation of funds and the encouragement to business innovation.

Both frameworks have been used for different purposes (e.g. the former to address system transformation, the latter to analyze public funding and venture capital), and sometimes in conjunction, e.g. regarding commercialization of public research: in the semantic space that we construct in this study, the concepts related to this mission (knowledge transfer and technology transfer) are positioned in-between the innovation systems thematic cluster and the one for business R&D. However, the finance thematic cluster is relatively isolated from the rest of the semantic set, suggesting that the systemic approach has not resulted in a transformation of the thinking around policy instruments used for STI itself.

Our analysis also illustrates an interesting interplay of permanent and changing themes. The systemic approach experienced several avatars as a response to new frameworks and new policy questions, but sometimes explained by following a changing policy lexicon. The industry-science relation also experienced several avatars, marking its progressive broadening from a one-way to a two-way exchange, from science and technology to all sorts of knowledge, from a specific higher education strategy to a transformative force at the core of the innovation economy (with the “knowledge triangle”). Hence, if changes in terms sometimes have a cosmetic (fashion) aspect, they can also convey information on actual evolution in the content or positioning of concepts. New policy demands have also emerged on the STI policy agenda including notably societal involvement in innovation policy, environmental matters but also digitalization.

Our paper contributes to the emerging set of studies that apply text mining tools, in which assessment is based on the implementation of standardized techniques on a shared corpus of text. It also illustrates how STI studies can provide a framework for evidence-based policy.

## Literature review

The analysis of STI systems has long comprised statistical analysis, performed with machines, and qualitative analysis, done by humans. It is only recently that, due to progress in natural language processing it has been possible to do policy analysis with the help of machines. This section presents a short review of the emergence and progress of semantic analysis in the STI policy field in a background dominated by statistical analysis.

One of the most widespread approaches in technological and scientific development studies is the analysis of publications and patent citation networks. This basic bibliometric method allows evaluating the impact of publications and authors on the field of interest. Furthermore, it indicates the ‘structure of the knowledge base on innovation’ using citation analysis and econometric tools (2012b; Fagerberg et al., 2012a). Patent analysis is a suitable tool for mapping technological trajectories (Verspagen, 2007). Martin (1995) provided an influential overview of foresight studies for STI which also demonstrated strong emphasis on bibliometrics and patent statistics. All these instruments form a basis for satisfying the needs of information based STI policy which was reviewed by Morlacchi and Martin (2009) in their seminal work and which also led to the emergence of innovation studies (Fagerberg, Verspagen, 2009). The analysis of field-specific highly cited publications allowed to draw a spectacular evolutionary panorama of science policy and innovation studies (De Silva et al., 2021; Gokhberg & Meissner, 2016; Martin, 2012).

Nowadays more detailed and in-depth results can be obtained by implementing advanced techniques making use of not only the metadata of publications but of their content, applying so-called semantic technologies, or text-mining. For instance, Zhang et al. (2017) adopted this approach and studied evolutionary pathways of scientific activities via textual data analysis, relationship identification models and science maps visualization. Science map construction was also applied by Shapira et al. (2017), who tracked the emergence of synthetic biology with their own bibliometric definition of this field. In broader terms, one of the highly dynamic topics in the scientific development analysis is identification of emerging trends, technologies and concepts. Among the most recent studies is the U.S. Intelligence Advanced Research Projects Activity Foresight and Understanding from Scientific Exposition Program (Carley et al., 2018) which published a software script for “Emergence Indicators” calculation for any topical issue. Important efforts in this field were undertaken by Daim et al. (2006), Chen (2006), Shen et al. (2010), Rotolo et al. (2015), Bakhtin et al. (2020), Gokhberg et al. (2020) among others contributing to the development of methodologies for emerging trends detection and consequently to its implementation in evidence-based STI decision-making. Semantic analysis is frequently used as a tool for identifying various types of particular STI trends in various areas.

Scientific research projects funding analysis is another popular area in STI policy studies where textual data analysis is regularly applied (Youtie et al., 2017). This is due to the fact that it provides an opportunity to enhance significantly the scope of information sources available, with big data arrays of unstructured textual content and financial data. For example, Huang et al. (2016) compared the US and China national funding of interdisciplinary big data research through text-mining processing of funded research proposals and funding acknowledgements of publications from the Web of Science database. Other valuable results in this sphere—cross-agency and cross-national co-funding patterns—were obtained by De-Miguel-Molina et al. (2017) who focused on the bibliometric analysis of scientific papers from the PubMed database and applied also Social Network Analysis methods.

Only recently semantic analysis techniques were exploited in this field, e.g. Zhang et al. (2014), who developed relevant methodology for the case of innovation in China’s dye-sensitized solar cell industry, or Li et al. (2018), who examined the influences on growth in small and mid-size firms within mentioned theoretical framework and using web-mining of companies’ websites.

Bakhtin et al. (2017) integrated different big data related information sources for trend monitoring of linking science and strategy. Another related theme studies with the help of textual data analysis instruments is scientific mobility. Here Kuzhabekova and Lee (2018) evaluated the influence of foreign university faculty members on Kazakhstan research capacity building by combining bibliometric, social network analysis, and content analysis of scholarly articles from the Web of Science database. Overall, the academic literature focuses exclusively on selected STI related themes and tends to ignore others. Furthermore, studies that scan policy documents and related analytical policy reports, besides scientific publications and patents, have been rather seldom, although using semantic tools and broader artificial intelligence solutions to mine relevant information could put policy debates on a sounder footing and help steer more-proactive policymaking (Gokhberg, 2020). Such attempts face several challenges so far:

1. National policy documents (including strategy papers, legal acts and analytical reports) differ substantially across countries in language (semantics, tone etc.) and focus, reflecting the national policy discussions.
2. Conversely, documents prepared by international organizations contain substantial cross-country information based on primary sources and provided to these organizations by national experts and framed in internationally harmonized or comparable language.
3. Eventually analysis of dedicated STI policy themes at a single country level leaves the overall STI policy mix consideration aside or covers it only partially meeting specific national circumstances.

Different from those cases, our analysis focuses on documents published by the OECD as an international organization that plays the key role in driving global cross-country discussions on STI policies and achieves general consensus between nations. The themes discussed there are not necessarily directly tied to concrete, immediate policy actions and programs, but rather to the overarching framework (objectives, issues, challenges, actors, tools) for policy initiatives and thus providing a broader—global—picture and understanding of the full range of STI policies.

The article continues as follows. Section 3 presents the data and methodology applied for document analysis, and Sect. 4 reports the major findings. The conclusions draw cross-cutting lessons.

## Data and methodology

### Data

This paper analyses a corpus of 210 documents of the WP TIP among which 95 are meetings and workshops agendas, 70 are meetings and workshops summaries and 45 are policy reports presented and discussed in particular meetings. These documents reflect 26 years of international discussions on STI policies at the WP TIP meetings from 1993 to 2019. The unique feature of these documents is that they reflect internationally harmonized compromise views achieved by participating nations in the framework of numerous cross-country projects and allied regular meetings. They are compatible with the national STI policies as their release requires consensual adoption by member countries representatives.

While the WP TIP documents are for the above-mentioned reasons a very suitable example to allow understanding how the global STI policy agenda evolved over nearly three decades, as is the case with any documents some issues reflect idiosyncratic developments specific to this international forum. For instance, the increased engagement of the European Commission in STI activities has reduced debates on international STI collaboration in the WP TIP that European countries address increasingly in the EU discussion fora. Consequently, the analysis of the results of semantic analysis requires expert adjustments to those idiosyncrasies. This reflected interpretation of results is done in the concluding section.

### Analysis process

**Fig. 1:**
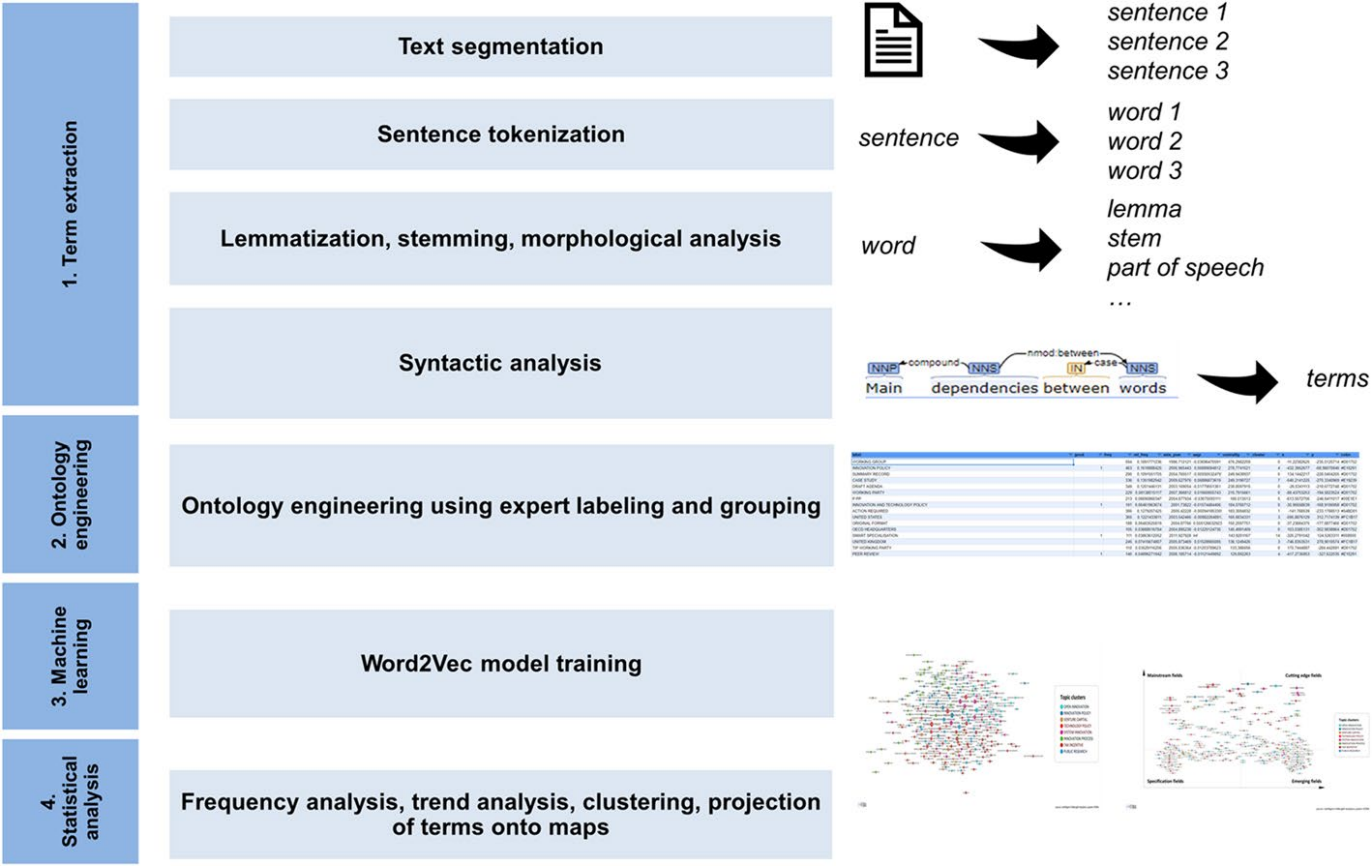
Stages and methods of analysis of the collected text array and related metadata. Source: HSE ISSEK iFORA

The process of the identification of main theme topics and changes over time was carried out using the Big Data *i*ntelligent *FOR*esight *A*nalytics (iFORA) system, developed at the HSE ISSEK.^3^ The system is based on natural language processing utilizing machine learning, text mining, science bibliometrics and technology analysis.^4^ Text data processing and analysis consists of four main layers (Fig. 1):

1. term extraction,
2. ontology engineering,
3. machine learning,
4. statistical analysis.

In the first stage (*term extraction* phase), large arrays of unstructured textual data were transformed into structured table representations. For achieving this, each document was split into separate sentences, words and phrases with different linguistic characteristics. Syntactic analysis of the links between words in the sentence allowed spotting word dependencies (where the first word is the governor, the second is dependent) and the type of communication based on the context of the interaction between words. The following dependency linguistic rules were used to iteratively combine all connected words into terms:

-  < *adjective, adjective modifier, noun* > (e.g. open innovation),
-  < *noun, compound, noun* > (e.g. venture capital),
-  < *noun, noun modifier, noun* > (e.g. internet of things).

Term extraction phase assumes that the universal numerical characteristics of words are calculated for the transition from the linguistic apparatus to the statistical and vice versa; this will allow separating the terms containing information about the object of study from the linguistic noise. The amount of literature devoted to the description of hybrid linguistic-statistical strategies that guarantee the extraction of reliable terms is growing from year to year, and testing the proposed methods for robustness when used on text corpora and various fields of knowledge, including technical texts, makes it possible to apply these methods within the framework of this study work. The most commonly used and effective measures for the selection terms are such statistical measures as word frequency, C-value and T-score, etc. Also, the high quality of the results is shown by such a measure as PMI, which is relatively simple log-linear function and is typical to use for neural networks to produce high-quality word embeddings on large corpora (Arora et al., 2016; Melamud & Goldberger, 2017).

In our context, based on results of computation PMI measure, approximately 3,000 terms were extracted from the texts. We extract 3096 words and cut the number to the closest round number for the further convenience. It can be noted that the increasing number of words lead to the decreasing governing statistics and some computational costs. Also, the vast majority have very general meaning when extracted from their context (e.g. “they have” or “text”), conveying no STI-related information that could be exploited with the current techniques.

In the second stage (*ontology engineering* phase), all terms were independently assessed, and only relevant policy instruments and topics were left for further analysis. We apply HCOME (Human Centered Ontology Engineering) methodology, involving the participation of linguists and experts in R&D policy, engineers, throughout the entire process, the effectiveness of which in projects from the field of medicine, R&D, business management, etc. is shown in a series of publications (Stapleton, 2006; Pereira et al., 2013; Hildebrandt et al., 2020). Details on design of ontology engineering phase can be found in Kotis and Vouros (2006). The 231 terms validated by experts in the previous step were clustered using hierarchical cluster analysis (see description in Murtagh & Contreras, 2012) into 168 higher level entities representing similar meaning (see Table 1 in Appendix A). For example, the following terms were grouped under “R&D TAX POLICY”:

- tax incentive,
- r&d tax incentive,
- r&d tax credit,
- tax treatment,
- tax credit,
- r&d tax policy,
- r&d tax treatment,
- tax relief,
- double taxation,
- tax system,
- tax base.

**Fig. 2:**
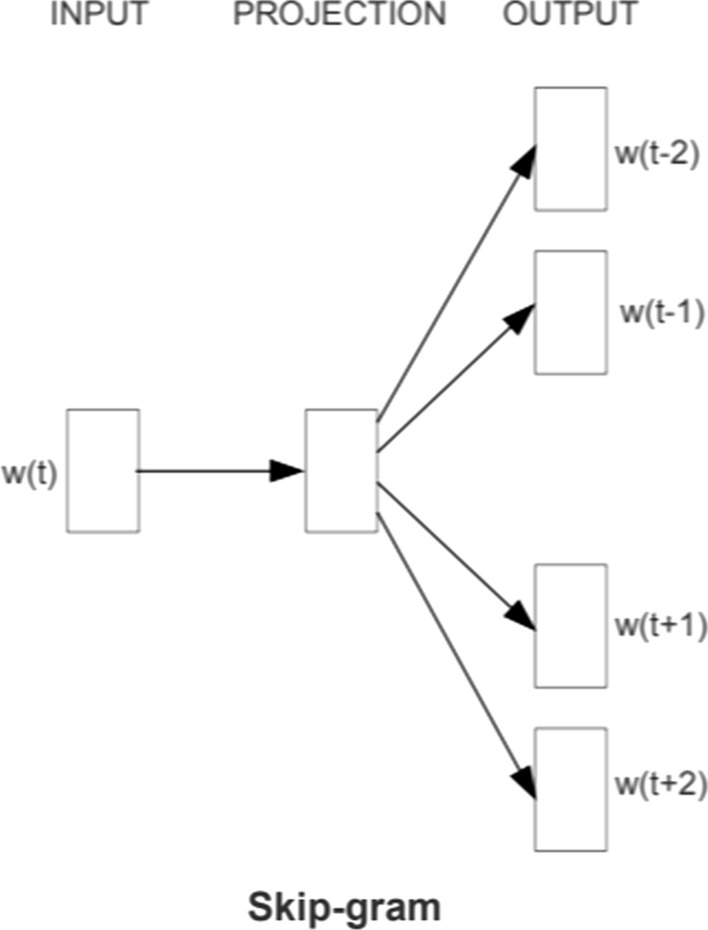
Word2vec Skip-gram architecture. Source: (Mikolov et al., 2013a)

Third, in the *machine learning* phase for the algorithm to learn terms’ meanings from WP TIP texts we applied a word embedding technique called Word2Vec (Mikolov et al., 2013a). The main idea behind Word2Vec is to discover word features based on the context of words (i.e. neighboring words). We used skip-gram shallow neural network architecture which aims at predicting context based on a word (Fig. 2**)**.

The main idea of the Skip-Gram architecture (parameter “Training algorithm”) is to maximize classification of a word based on another word in the same sentence. Word prediction is carried out using an iterative procedure, where the input to a log-linear classifier with continuous projection layer is a current word, for which we determine the size of the interval where word representations should be estimated (parameter “window size”—maximum distance between the current and predicted word within a sentence). An increase in the size of the interval leads to a significant increase in computational complexity, however, it has a positive effect on the quality the resulting word vectors.

In Skip-gram model for the task of finding word representations we take a sequence of training words ${w}_{1}, {w}_{2}, {w}_{3}, . . . , {w}_{t}$ and try to maximize the average log probability$\uprho$:

1 $$\frac{1}{T}\sum_{t=1}^{T}\sum_{-c\le j\le c,j\ne 0}\mathrm{log} \rho \left({w}_{t+j}\left|{w}_{t}\right.\right)$$

C—window size, ${w}_{t}$—input word, T—vector size (parameter “word embedding size”).

In (Mikolov et al., 2013a) it is proposed to compute $\mathrm{p}\left({w}_{t+j}\left|{w}_{t}\right.\right)$ on the base of softmax function

2 $$\uprho ({w}_{O}|{w}_{I}) = \frac{exp({{\nu {^{\prime}}}_{{w}_{O}}}^{{\rm T}}{\nu {^{\prime}}}_{{w}_{I}})}{{\sum }_{w=1}^{W}exp({{\nu {^{\prime}}}_{w}}^{{\rm T}}{\nu {^{\prime}}}_{{w}_{I}})}$$

${w}_{I}$—input vector representations of w, ${w}_{O}$—output vector representations of w, W—number of words in the vocabulary.

Such formulation is time-consuming and non-effective in term of computer calculations, it is better the well-known approximation Hierarchical Softmax. It is first introduced by Morin and Bengio (2005) especially for the tasks of neural network language models. Hierarchical Softmax allows to decrease computational cost by evaluating only about ${log}_{2}$ W nodes to obtain the probability distribution. More details about the procedure can be found in (Mikolov et al., 2013b). Hierarchical Softmax approximation does not apply together with Negative Sampling, it operates only with high-quality vector representations (parameter “Negative sampling”).

Skip-Gram architecture is applied to the tasks from a wide range of scientific and applied problems in neural networks for sentence classification and show the robustness of the results to the parameter settings, and the advantage of the architecture over other analogues, including CBOW.

To train the Word2Vec model we used Gensim software with the following parameters:

**Table:** 

Parameter	Value
Training algorithm	Skip-gram architecture
Softmax function approximation	Hierarchical softmax
Window size (maximum distance between the current and predicted word within a sentence)	15 (in words)
Word embedding size (dimensionality of the vector representations of words)	200
Minimum count of words (ignores all words with total frequency lower than this)	3
Number of iterations	10

Thus, the above specification of the architecture and the choice of parameters are aligned with generally accepted practices for the implementation of these algorithms and take into account the need to balance the accuracy of the results and computational overhead. The robustness of the architecture itself is confirmed by the publication of materials with the results of comparative testing of algorithms.

The key trick in the *machine learning* stage was to use terms extracted in the first phase of the analysis instead of words, and to account for higher level entities. In order to achieve that the following transformations were made:

- words contained in terms, that were detected inside sentences, were combined together using “_” symbol. For example, the sentence “Triple helix is an important concept in STI policy” would transform intro “***triple_helix*** is an ***important_concept*** in ***sti_policy***”,
- terms that were grouped under one entity during the ontology engineering phase, would be replaced by their respective entity.

Finally, in stage 4 the *statistical analysis* was performed in order to derive trends from the data. Two main metrics were calculated:

- relative position of terms,
- relative term frequency,
- term growth rate.

The annual relative term frequency ${tf}_{i}(t)$ was calculated using the following formula (3):

3 $${tf}_{i}(t)=\frac{|\left\{{s}_{i}\in {S}_{i}|t\in {s}_{i}\right\}|}{{|S}_{i}|} ,$$

$|\left\{{s}_{i}\in {S}_{i}|g\in {s}_{i}\right\}|$—the amount of times the term $t$ occurs in the sentences in documents of a particular *i*th publicatio*n year,*

${|S}_{i}|$—the amount of all *o*ccurrences in all sentences in all documents of a particular *i*th publication year.

The total relative term frequency $tf(t)$ was calculated using the following formula (4):

4 $$tf(t)=\sum_{i=1}^{T}{tf}_{i}(t) ,$$

$T$—the length of time period in years, $i=1, 2,\ldots ,T$

The following indicator helps to assess the significance of the topic in a large number of documents.

Term growth rate $gr(t)$ was calculated using the following formula (5):

5 $$gr(t)= \frac{1}{T}\left(\frac{\sum_{i\ge T/2}{tf}_{i}(t)}{\sum_{i\le \frac{T}{2}}{tf}_{i}(t)}-1\right) ,$$

$T$—the length of time period in years, $i=1, 2,\dots ,T$,

${tf}_{i}(t)$—annual relative term frequency in *i*th publication year.

Term growth rate allows spotting trends in the data—the higher the overall dynamics, the more potential this topic may gain in time.

The results of the statistical analysis were visualized in the format that would be intuitive and insightful for potential decision makers, researchers and experts. To accomplish that we used *semantic (wordcloud)* and *trend maps*.

**Fig. 3:**
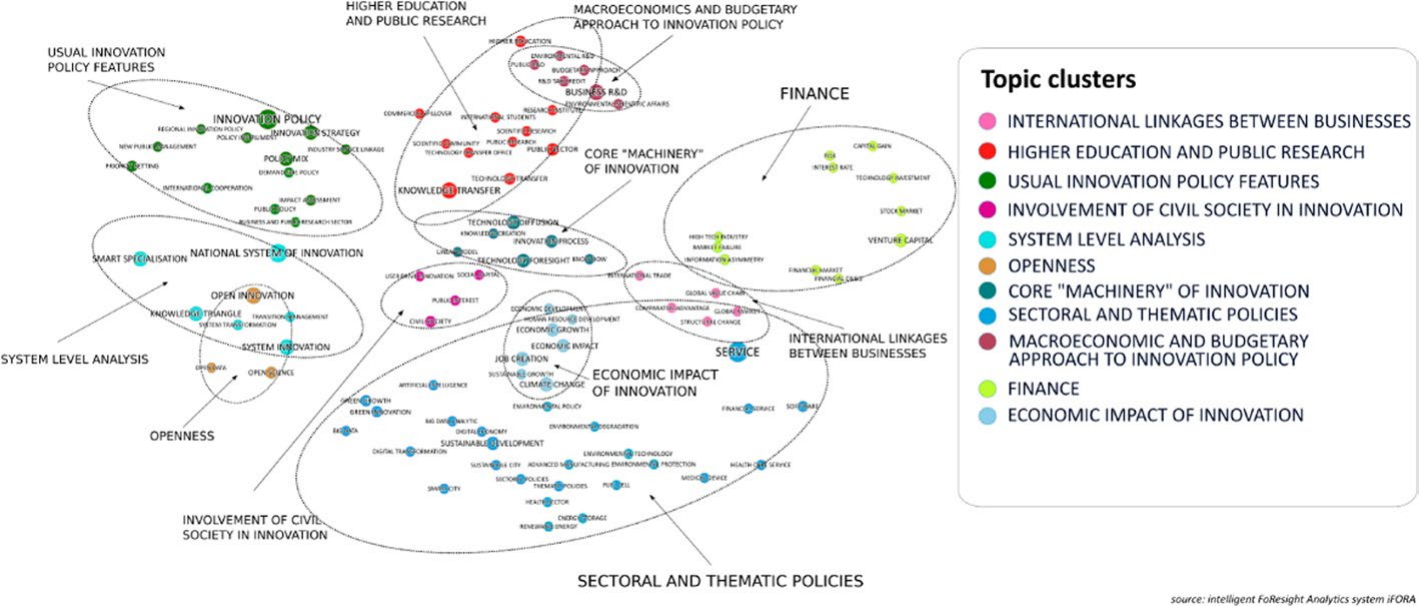
Semantic map of WP TIP topics, 1993–2019. Source: authors based on iFORA, HSE ISSEK

1. *Semantic map* is a projection of terms’ vector representations (Word2Vec word embeddings) on a two-dimensional plane, where:
  coordinates of terms are based on the vectors’ projection that builds upon using UMAP algorithm (McInnes et al., 2018) with following parameters:
    set of parameters which can be used for balancing local and global structure of the nodes and links on the map:
      number of approximate nearest neighbors used to construct the initial high-dimensional graph = 20 (the greater the value of the parameter is, the more common features will be presented on the map, the lesser the role will be played by local or rare connections between nodes),
      minimum distance between points in low-dimensional space = 0.6 (the larger value is the less focus on the broader topological structure takes place. This parameter controls the density of links),
    metric (mathematical representation of the distance between nodes) = cosine,
    number of components (construct topological spaces out of simple 2-simplex components; *k*-simplex as an arbitrary set of *k* + 1 objects with faces) = 2.
  term size is determined by total relative term frequency,
  color is specified by cluster, which was identified using *Ward hierarchical clustering* algorithm (Ward, 1963) based on terms’ coordinates,
  each cluster of terms—is a separate topic unified semantically.
2. *Trend map* is a scatter plot, which allows identifying trends of different types based on combining two metrics: *total relative term frequency* (2) and *term growth rate* (3). By using median values of both metrics, we came up with 4-level classification of all terms represented by following classes. The principal scheme of *trend map* is demonstrated on the Fig. 3. The color of terms was determined by thematic clusters assigned on semantic map. Instead of pure *term relative frequencies* and *term growth rates* we used their *inverse ranks* in order to normalize their distribution on the map (for better readability): such a transformation changes the levels of the variable but preserves the ranking of the terms. The size of labels of terms would still refer to real values.

Thus, the methodology described above is in line with accepted practices in the scientific community for conducting research in the field of NLP. This work refers to a practice-oriented study, without focusing on a significant development of the methodology, but rather the application of a highly cited and relevant methodology to the task of strategic planning and studying the WP TIP policy agenda. A sketch of the methodology is intended to provide background information for the convenience of the reader, and also provides the necessary links for a more detailed study.

### Caveats to the analysis

While semantic analysis is a powerful tool, the following caveats apply. First, judging the relative importance of the various themes on the WP TIP policy agenda is not straightforward on the basis of semantic analysis only. Although counts of terms are informative, they are not sufficient: because terms have uneven semantic scope (e.g. “international cooperation” is broader than “international technological cooperation”), and because terms have to be connected to each other in order to identify themes or broader thematic clusters. More sophisticated methods could capture semantic relations between terms and control for such effects. This was done here partly manually (e.g. by grouping certain terms considered as absolute synonymous), but to a limited extent as all manual interventions carry the danger of reflecting the researcher's own prejudice (see examples of ambiguous relations between terms below).

Second, changes in terms could either reflect changes in the semantic content or could just be cosmetic changes, as terms can be subject to some types of academic or political fashion. The distinction between the two possibilities is not always clear-cut, as formal changes, in relation with some fashion, can also denote new nuances in the semantic content. For instance, the word “technology” has often been replaced by the words “knowledge” or “innovation”, depending on the context. Hence, “technology transfer” has been replaced by “knowledge transfer”, and “technology policy” by “innovation policy”. While the meaning can be unchanged in certain cases, this change from “technology” to “knowledge” or “innovation” can also denote a broadening of or even change in the focus—knowledge is broader than technology; innovation is about a specific economic operation or cycle while technology refers to a technical phenomenon. Hence, “technology transfer” has the connotation of a purely technical operation, while “knowledge transfer” might refer to economic, managerial and cultural dimensions, as well.

Third, overall, the analysis shows that thematic areas overlap and interrelate, but no causality can be derived from this. Interpreting the findings requires substantial expertise and experience. The statistics captures the occurrence and co-occurrence of terms in documents and discussions but this does not reflect the whole semantic dynamics. For that purpose, one needs to constitute wider policy themes by grouping the terms, one needs to analyze the connections between these groupings, and above all one needs to embed this evidence into a broader knowledge of the corresponding policy debates. Despite the methodological progress provided by text mining techniques, there is still much human intervention left in such an analysis. Consequently, the section of findings adds those framings to the results of semantic analysis to jointly produce a new vision on the developments of STI policies.

## Findings

This section discusses the findings from implementing the above-mentioned methodology to address what have been the main themes of the global STI policy agenda represented by the WP TIP as well as stability or change in those themes over the 1993–2019 period.

### Main policy themes

To uncover the main policy themes and their linkages, we produce a semantic map of the WP TIP documents as shown in Fig. 3. Each term can belong to several different topic clusters—the classification is not exclusive. The proximity between words on the map is reflective of their spatial proximity (co-occurrence) in the WP TIP documents. In most cases, this is because the terms feature in a same WP TIP project, which is directly reflective of contextual, semantic proximity, i.e. the closeness of terms in a same STI policy sub-domain. Proximity could also emerge from different projects that were conducted at the same time and present in contiguous items on the WP TIP agenda. While not necessarily reflective of a semantic link, this proximity is informative of the “mood of the time” regarding the STI policy discussion. In view of these observations, the semantic map derived from our iFORA-based experiment indicates the following automatically identified main STI policy themes (topic clusters):

- International linkages between businesses
- Higher education and public research
- Usual innovation policy features
- Involvement of civil society in innovation
- System level analysis
- Openness
- Core ‘machinery’ of innovation
- Sectoral and thematic policies
- Macroeconomic and budgetary approach to innovation policy
- Finance
- Economic impact of innovation

These STI policy themes are described in the following.

**International linkages between businesses**: *international trade, global value chain, global market*. These terms are close on the map to “*comparative advantage*” and “*structural change*”, which are important economic concepts: the latter notably reflects one expected impact of international trade and investment on national economies. At the same time this cluster is separate from terms like “*international cooperation*” (which is included into the *usual innovation policy features* cluster) and “*international students*” (which goes together with *higher education and public research*): that might reflect a weak integration of business and public sector dimensions in the policy mix for internationalization as viewed by the WP TIP.

**Higher education and public research**: *research institute, scientific research, public research, public sector, scientific community, higher education, international students, knowledge transfer, technology transfer, technology transfer office*, *commercial spillover,* etc. This thematic cluster gathers science-related institutions with public missions and government funding, and their strategic orientations. *Commercialization* has become a major mission of higher education institutions over the years. It is therefore not an accident that this cluster borders with both *business R&D* and *technology diffusion* related topic clusters on the semantic map. On the other hand, it is quite far from the “*knowledge triangle*”. The latter is used in a different policy and semantic environment, related to broader *system* approaches rather than higher education policy per se.

**Usual innovation policy features**: *demand side policy, public policy, business and public research sectors, industry-science linkage*, *innovation policy, innovation strategy, regional innovation policy*, *priority setting, new public management,* etc. This is a set of instruments that are used in a coordinated way (“*policy mix*”) to constitute the innovation policy toolkit. In many respects it is the core mission of the WP TIP, to analyze the design and implementation of these instruments for different purposes, in different contexts, to different objectives. This thematic cluster is close to the “*system level analysis*” and “*higher education and public research*” clusters, more than to the *macroeconomic* and *finance* clusters: this might reflect a certain orientation of the WP TIP views giving more weight to the corresponding conceptual approaches and instruments.

The **involvement of civil society in innovation**: *civil society, public interest, social capital, user-driven innovation*. This set of themes is rather recent as we will see, it reflects the emerging trend across countries for new channels (beyond markets and governments) to influence the orientation of innovation, so as to take into account considerations and objectives that existing channels have tended to underestimate (in relation with various aspects of individual or collective well-being like health, the environment etc.).

**System-level analysis**, looking at the various actors and their interactions, embodied in terms like: *National System of Innovation (NSI), system innovation, system transformation, transition management, smart specialization,* and *knowledge triangle*. These terms refer to broad concepts that have been used by the international STI policy community to characterize major trends in the economy and policy of innovation. Interestingly in all of these cases, the central idea is about linkages between actors, used to leverage some policy objective: this is reflective of a systemic view of innovation. Each theme has been the subject of at least one dedicated WP TIP project at some point in time. In the case of the *NSI*, the TIP played a significant role in the development of the concept in its early years (1990s), and applied it widely for more than a decade subsequently. Each other term in the list points to a specific phenomenon having a broad impact: *system transformation* is a specific approach to the dynamics of adapting societies to societal and environmental challenges; *smart specialization* focuses on the regional dimension of innovation-based development; the *knowledge triangle* is centered on higher education institutions and their contribution to innovation. System-level analysis tends to put as much or even more emphasis on non-market relationships between actors as on market relationships, to emphasize the role of institutions, the embeddedness of economic development in societal networks and the specific features of human communities at various scales—regional or national.

**Openness**: *open innovation, open science, open data*. Access to skills and knowledge is key for innovation, and the way it is structured has a direct impact on innovation performance. The years 2000s have experienced a dramatic change in that regard, with the progress of the Internet and digital technologies, which have reduced the cost of communication and increased its density. New opportunities arose for circulating information, but their realization required certain policy conditions to be met. This is what the WP TIP discussed in the fields of data, science and innovation.

The **core “machinery” of innovation**: *knowledge creation, innovation process, know-how, technology diffusion, technology foresight, linear model*. These terms reflect various aspects on the generation and diffusion of innovation. In order to strengthen its policy analysis, the WP TIP has investigated these deeper aspects of the innovation system, with a view notably to establishing a “post-linear” model of innovation. The *linear model* states that science precedes technological innovation, which precedes marketization; while the alternative models insist on feed-back linkages in this chain. For instance, the linear model which is still present in many national strategic documents across the globe tends to justify a separation of innovation policy from science policy, an approach opposite to the systemic view, according to which actors and activities are closely interconnected and such separations are detrimental to the efficiency of the system.

**Sectoral and thematic policies**: digital (*artificial intelligence, Big Data, software, digital transformation, digital economy*, etc.), *services*, specific technologies (*advanced manufacturing, fuel cells, energy storage, renewable energy, smart city*), and specific social needs (*health sector*—*health care services, medical devices*, environment—*environmental policy, environmental degradation, environmental protection, sustainable development, green growth, green innovation, sustainable city*). These dimensions are strongly interconnected, the corresponding terms tend to occur in the same documents, demonstrating that the WP TIP has taken a broad, holistic view of these issues, emphasizing their generic aspects in relation with innovation: sector specific aspects were enlightened with broader considerations like technological transformation (digital) and social demand (health and environment). Interestingly in that regard, two policy terms connected with environment (*environmental R&D* and *environmental scientific affairs*) are not in this cluster but rather in the proximity of the policy instruments they use (R&D and science respectively), showing that the WP TIP has been working to connect social demand with the core policy instruments of its domain.

**Macroeconomic and budgetary approach to innovation policy**: *business R&D, public R&D, R&D tax credit, budgetary approach, environmental R&D, environmental scientific affairs*. The WP TIP has complemented the systemic analysis with economic analysis, investigating the structure and impact of R&D spending (one of the major sources of innovation, for which reliable statistical data is available) and the policies that can influence it. It has conducted several quantitative studies on the *R&D tax credit* instruments, that were embraced by a dozen of countries in the mid-1990s, at the WP TIP’s inception, and appeared again in an exercise implemented by nearly all (36) OECD member countries in 2019. The conceptual framework for doing that is complementary from the systemic approach, it relies more on mainstream economic concepts like rate of return, optimization, etc.

**Finance**: *venture capital, stock market, technology investment, interest rate, capital gain, financial market, financial crisis, high tech industry*. This thematic cluster is close to the “*macroeconomic and budgetary approach*” cluster mentioned above. Both have to do with resource allocation, the former by firms and markets, the latter by government. This domain makes use of economic concepts, like *risk* and *information asymmetry*. The main justification for government intervention in a classical economic framework is the existence of *market failures*: this term is linked to *high-tech industries*, a sector whose development raises complex financing issues related to *risk* and *information asymmetries* (as it is the main recipient of venture capital) and where government is active due to alleged market failures.

**Economic impact of innovation**: *economic growth, economic development, sustainable growth, job creation, human resource development*, *climate change*. Governments allocate significant resources to innovation (including R&D), and want to know how effective this effort is, notably in terms of *growth* and *job creation*. Consequently, this has been a major issue of the WP TIP, and an important component in many STI policy projects.

Overall, tracing the activities of the WP TIP over the past decades points to two separate but complementary policy frameworks:

1. a structural, systemic one, emphasizing the linkages between actors on the one hand, and
2. an economic, budgetary one focusing on the allocation of funds and the encouragement to business innovation on the other hand.

They have been used for different purposes (e.g. the former to address system transformation, the latter to analyze venture capital), and sometimes in conjunction, e.g. regarding the commercialization of public research: the terms reflecting this mission (*knowledge transfer* and *technology transfer*) are positioned in between the innovation policy domain and the business R&D field. However, the *finance* thematic cluster is relatively isolated from the rest of the semantic set, suggesting that the systemic approach has not resulted in a transformation of the thinking around policy instruments used for STI itself. Also, Intellectual Property Rights (IPR) are close to no specific cluster or set of topics: that might reflect the fact that IPR is a very transversal, cross-themes topic, used in various contexts like *international trade*, *technology diffusion, technology transfer, industry-science linkages*, *innovation policy*, *sectoral policies,* etc. This is one limit of the semantic technique, since it cannot reflect the variety of contexts in which a particular term is used, but it just averages them.

### Stability and change in the WP TIP thematic priorities

**Fig. 4:**
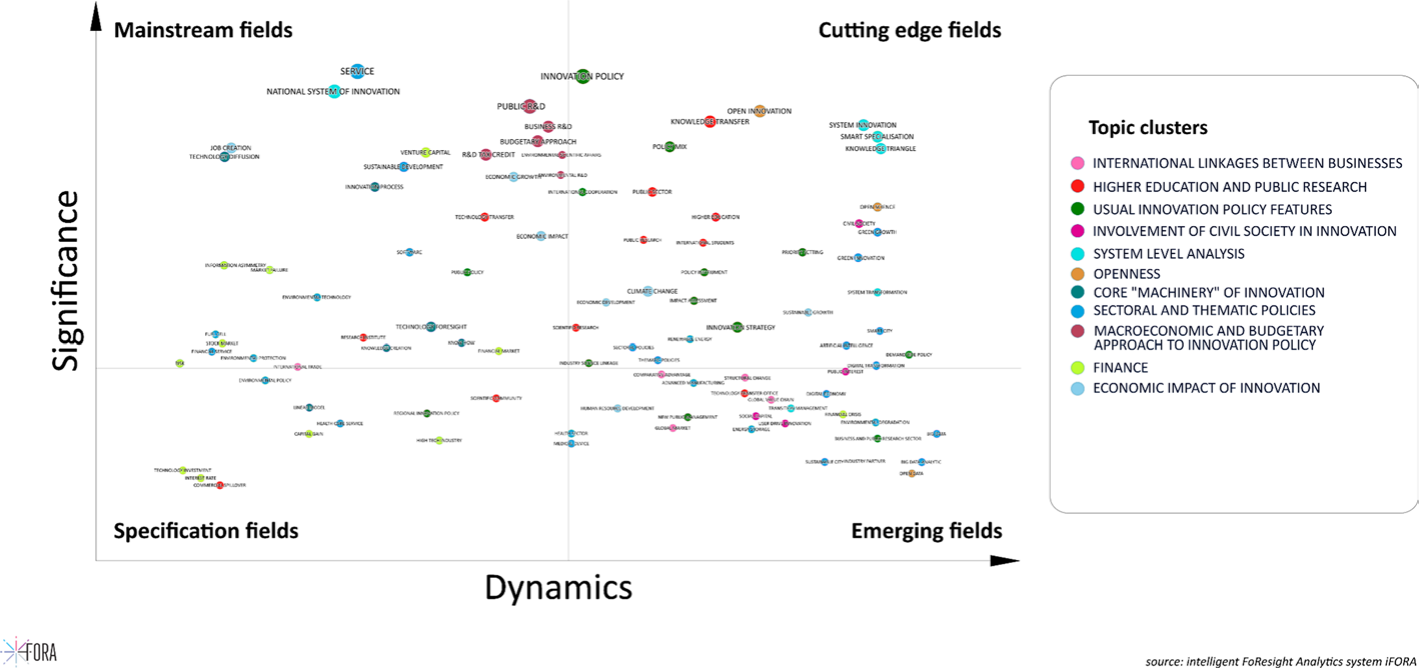
Trend map of WP TIP topics, 1993–2019. *Source* authors based on iFORA, HSE ISSEK

We now turn to understanding how the WP TIP thematic priorities evolved over the 1993–2019 period. The vertical axis of Fig. 4 reflects the relative frequency of terms’ appearance over the entire time period, while the horizontal axis demonstrates the change in frequency over the periods Hence terms positioned in the north-east quadrant are both highly significant and in relative progress over time, while the ones resting in the north-west quadrant remain influential under the current mainstream but eventually losing momentum. Terms located in the south-east quadrant represent emerging STI policy fields gaining greater dynamics, and those attributed to the south-west quadrant are weak signals with a promise of further growth. We did not make an explicit analysis of the evolution of terms during specific sub-periods, namely the 2008 financial crisis and the 2012 EU budgetary crisis in Greece. The reason is that the 2008 financial crisis did not impact the discussion at the Working Party much. This is mainly due to the overall modest impact of the crisis on R&D spending during this period. Following a short dip in spending in 2009 related expenditures recovered quickly and continue to grow since then (OECD, 2009; Rehm, 2018). Certainly, the picture was different during this period where countries with traditionally strong innovation performance (namely the Nordic countries, Germany, Switzerland, the US) faced rather low decrease while other countries (Southern and Eastern European countries) experienced stronger decrease of R&D expenditures. The latter however were rather quickly offset with funds from the European Union (structural funds) (Rehm, 2008). In addition, it was evident to the WP TIP that R&D expenditure follows a mid to long term planning schedule which is usually not affected by short term events (Alfranseder, Dzhamalova, 2014). Kincso and Radosevic (2017) argue that the crisis has led countries to reconsider their STI policy mix composition by means of streamlining the number of policies in place and supporting co-funding of business R&D expenditure by public funds which aims at replacing otherwise decreasing volume of private funding due to financial constraints (Peia and Romelli, 2022). In the end, the 2008 financial crisis did not seriously affect R&D on broader scale industry-wide but did impact mainly those companies which were already under financial constraints anyway, e.g. companies who had to restructure their own finance towards short-term meeting financial obligations. Therefore, the impact topic was not at the WP TIP agenda explicitly and thereby not affecting issues discussed. Instead, the agenda continued as usual. Although there are special developments in selected countries one has to remember that the OECD WP TIP includes countries from all around the world, not limited to regions. Therefore, specific regional challenges are not an issue of discussion but the overall global picture is. This also explains why this paper does not split the sample according to events which occurred during the period under investigation.

The figure provides the evidence of a substantive change. The *NSI*, a concept central to the WP TIP original DNA, has experienced a sharp decline in policy use. In reality, this does not mean a decline in the application of the systemic approach, as the notion of *system innovation* remains among the top terms. Rather it reflects growing awareness that the sub-national level (with *smart specialization*) and the supra-national level (with *global value chains* and *international cooperation*) have become more important relative to the national level. It is also explained by the emergence of substitute terms like *system transformation* or *knowledge triangle*, which in certain respect reflect the *NSI* approach for particular applications—hence reducing the need to directly use the term *NSI* but not signaling the demise of the systemic approach role in the STI policy discourse.

Three policy concepts feature prominently among the terms that experienced high growth in use: *smart specialization*, *system innovation* (or *transformation*), and the *knowledge triangle*. These STI policy concepts are popular notably among the EU policy makers and have been subjects to dedicated WP TIP projects involving experts from European countries and the European Commission. *Smart specialization* has been promoted by the European Commission as an instrument for making innovation a driver of economic growth at regional level. It is based on systemic principles, emphasizing the market and non-market connections between actors and between economic activities, and attempting to leverage those linkages in order to support collective learning dynamics within regions. As for *smart specialization* and *system transformation*, the WP TIP documentation analysis demonstrates that these policy approaches are actively promoted by Nordic countries which have triggered interest of others. Also based on systemic principles, they ambition to leverage linkages in the *NSI* in order to diffuse innovations across the economy and exploit dynamics that are nurtured in specific segments—the higher education system for the *knowledge triangle*, or particular sectors like energy or transportation for the *system transformation*.

Looking at the usual innovation policy features, the terms *technology diffusion* and *regional innovation policy* have declined, while *innovation policy* has been kept at the top. Like mentioned above, this reflects not only just a cosmetic change, but also a broadening in the scope of innovation and innovation policies. Innovation is no longer seen as just technology implementation, it concerns new ways of delivering services, new business models and marketing strategies; social and environmental innovation have also become focal areas of public policies and corporate strategies. Accordingly, innovation policies are not conceived anymore as just targeting technology per se but all its environment, and they have also to do with most aspects of the economy: education, competition, trade and so on—in other words *technology diffusion* and *technology foresight* remain on the agenda but mainly in the context of innovation. Having said this we mean that technology is seen as a silo notion and *innovation* has determinants and impacts all across the economy and society. This is further evidenced by the fact that most terms featuring the word “*technology*” have declined in fact (*technology transfer, technology diffusion, technology investment,* etc.).

References to *international cooperation* have also significantly decreased, although the phenomenon has grown in importance in the global economy. One prominent WP TIP project addressed *global value chains*, which have to do with businesses: but most policy projects have focused exclusively on the national and regional levels, and *international cooperation* have seen its priority reduced on the WP TIP agenda. This is not a general phenomenon in the field of policy analysis, but specific to this international expert group: the growing role of the European Commission, which manages the largest program in the world for international cooperation in R&D (the Framework Programme) could partly explain that the WP TIP experienced a weaker involvement in this matter, in view of not duplicating activities implemented elsewhere.

By contrast, some of the most prominent terms have remained at the top, reflecting stability in their importance and probably in their meaning, including *public R&D* and *business R&D*. *R&D tax credits* diffused to more and more countries and increased their financial importance. As for *public R&D* and *business R&D*, these are central activities without which no innovation policy can be conceived.

Figure 4 illustrates also the emergence of a more strategic and integrated way of designing and implementing innovation policy: this is reflected in the rise of terms like *innovation strategy*, *priority setting*, *impact assessment*. The WP TIP work played a central role in the “OECD Innovation Strategy” that was published in 2010. This very much mirrored the mainstreaming of innovation across all policy domains. The WP TIP devoted several projects over recent years to various aspects of *impact assessment*, a theme of growing interest in countries where budgetary conditions command stronger surveillance of the efficiency of public spending. At the same time, the term *technology foresight* relatively declined, possibly because of the recognition that innovation policy should be less focused on technology per se, even sometimes technology neutral (the “end of pipe” approach). This may be changing with the 2020 COVID-19 pandemic crisis with concepts of technology resilience appearing on the policy agenda although not shown in the analysis yet.

The terms *service* and *software* have moved outside the cutting-edge fields, probably being replaced by *digital economy* terms, which are more adequately reflective of the growing importance of the digital technologies in innovation matters. It is illustrated by the fact that terms associated with new modes of knowledge sharing, linked with digitalization, are on the rise: *open innovation, open science, open data*. The WP TIP has addressed the digital transition through several projects over the years, focusing on its impact on science, innovation and the economy.

New concerns of the 2010s gave birth to integrating such concepts as *civil society* (that should be stronger involved in policy design and implementation), or *green growth* and *climate change* (new structuring themes for policies), into the emerging global STI policy agenda. The WP TIP implemented a study on *green innovation* in the late 2000s already and has kept active in this area since then.

## Discussion and conclusions

An important feature demonstrated by our analysis is the dominance of a systemic approach to innovation; initially embodied into the *NSI*, later morphed into *system transformation* and *smart specialization*. One can suspect that with the COVID-19 pandemic crisis, further emphasis will be set on *system transformation* as narrower economic approaches are considered not to be paying enough attention to systems interdependencies and shocks.

A second feature is a permanent focus on industry-science relationships, under various successive labels like the commercialization of *public research, technology transfer, knowledge transfer* or more recently the *knowledge triangle* and co-creation.

A third feature, that is visible in the two first findings, is the adaptation of permanent impactful themes to a changing context. The systemic approach experienced several avatars as a response to new frameworks and new policy questions. Industry-science relationships also experienced numerous repaints, marking its progressive broadening from a one-way to a two-way exchange, from science and technology to all sorts of knowledge, from a specific higher education strategy to a transformative force at the core of the innovation economy (with the *knowledge triangle*). Hence, if changes in terms sometimes have a cosmetic (fashion) aspect, they can also convey information on actual conceptual changes. A well-developed methodology for evaluating terms, including expert validation of the results obtained, makes it possible to differentiate cosmetic changes in STI policy from really significant policy transformations, which makes the presented results reliable for further use by decision makers.

A fourth feature is the emergence of new policy demand and the embrace of new frameworks: societal involvement in innovation policy, climate and environmental matters, more recently digitalization. The WP TIP developed specific analyses after the financial crisis of 2008, looking at the analytical knowledge and historical record regarding the impact of the economic cycle on innovation.

Overall, these results are partial but encouraging for the potential of semantic analysis in studying STI policy. The analysis presented above suggests already that the WP TIP themes are largely reflective of the status of nearly three-decade long discussions in the global innovation policy community at large, both in terms of issues to be addressed and in terms of conceptual frameworks to rely upon. Since the corpus of analysis is limited by the WP TIP documents results need to be understood explorative thus not fully representative and generalizable. Therefore a next step could be to apply semantic techniques to a broader corpus of innovation policy related documents, including for instance other OECD bodies, also non-OECD international and national agencies, and academic literature. A promising area to discover is the comparison of policy and academic narratives for STI. That would allow drawing a broader and deeper picture of the debates in the STI policy arena over the past decades, hence identifying a gap between the academic world and policymaking practices for the sake of both communities. It can be assumed that there is a time lag between the academic discussion and the uptake of themes in the policy arena whereas the themes as such do not differ substantially. This is explained by the fact the WP TIP like most other international Working Groups or Committees are frequently inviting academics to their meetings to get fresh inputs from the academic world or involve academics in the preparation of scoping documents. Publications of these groups often include a substantial information on countries national (federal and regional) STI policies which are used by academics for further analysis thereby influencing the academic discourse. For example, OECD publishes the biannual STI Outlook which is a standard widely accepted publication including a collection of recent national STI policies information in a structured way, which makes them more comparable across countries. It is notably the aim of the OECD STIP Compass, which is a companion database made in cooperation with the European Commission. Since countries, namely national / federal ministries, provide information first hand for these publications and databases, policymakers somewhat influence the stream of academic research that uses this information. Thus, there is a close connection between policymakers and the academic community which in turn makes it little surprising that the main themes of STI policy remain similar over the period analyzed. A major input from academia to policymaking appeared with the NSI as an analytical concept at the last decade of the twentieth century and the following focus on STI policy instruments under the NSI framework. Until recently a strong orientation of academic STI policy studies towards the institutional framework and linkages inside the framework is observed.

## Appendix

See Table 1.

**Table 1:** Topics from the OECD WP TIP discourse with indicators

Label	Term frequency	Relative term frequency	Axis year	AAGR of relative frequency	Cluster	quadrant	GOOD
ADVANCED MANUFACTURING	11	0,210,956,893	2009	0,031,668,729,609,233	16	Emerging fields	1
ARTIFICIAL INTELLIGENCE	16	0,231,475,811	2017,6875	0,53,462,157,809,984	11	Cutting edge fields	1
AUTONOMOUS VEHICLE	9	0,15	2016	Inf	11	Emerging fields	
BIG DATA	5	0,105,231,254	2013,8	Inf	11	Emerging fields	1
BIG DATA ANALYTIC	5	0,076,489,533	2018	Inf	11	Emerging fields	
BUSINESS AND PUBLIC RESEARCH SECTOR	2	0,028,169,014	2008	Inf	5	Emerging fields	
BUSINESS INNOVATION	47	0,911,500,245	2008,042,553	0,0,142,638,807,101,159	10	Cutting edge fields	1
BUSINESS INVESTMENT	3	0,041,131,291	2002,666,667	− 0,0,177,501,445,107,079	6	Specification fields	1
BUSINESS MODELS	30	0,588,998,895	2013,733,333	0,754,887,333,298,225	13	Cutting edge fields	1
BUSINESS R&D	147	2,788,776,774	2005,870,748	− 0,0,110,091,205,850,478	10	Mainstream fields	1
BUSINESS SECTOR	46	0,748,478,459	2005,173,913	0,00,107,148,168,169,841	10	Cutting edge fields	1
CAPITAL GAIN	9	0,125,195,618	1997,333,333	-0,0,323,422,013,562,859	7	Specification fields	
CIVIL SOCIETY	61	0,92,530,966	2017,639,344	2,53,050,126,534,455	8	Cutting edge fields	1
CLIMATE CHANGE	28	0,476,121,535	2010,5	0,297,955,705,589,179	16	Cutting edge fields	1
CLUSTER ANALYSIS	7	0,11,162,419	1995,714,286	-0,037,037,037,037,037	1	Specification fields	
COLLECTIVE INTELLIGENCE	4	0,057,971,014	2019	Inf	11	Emerging fields	1
COMMERCIAL SPILLOVERS	3	0,045,546,559	2004,333,333	-0,037,037,037,037,037	3	Specification fields	1
COMPARATIVE ADVANTAGE	20	0,360,414,861	2008,4	0,12,986,437,616,324	13	Cutting edge fields	
CRITICAL TECHNOLOGIES	21	0,348,868,971	2005,142,857	− 0,0,141,254,125,412,542	1	Mainstream fields	1
DEMAND-SIDE POLICIES	16	0,253,592,412	2008,8125	Inf	5	Cutting edge fields	1
DIFFUSION PROCESS	5	0,083,333,333	1996,4	− 0,037,037,037,037,037	13	Specification fields	
DIGITAL ECONOMY	11	0,292,997,543	2015,454,545	0,403,403,403,403,404	11	Emerging fields	1
DIGITAL TRANSFORMATION	12	0,224,050,137	2018,333,333	Inf	11	Emerging fields	1
ECONOMIC ASSET	4	0,076,923,077	2005	− 0,037,037,037,037,037	15	Specification fields	
ECONOMIC DEVELOPMENT	33	0,61,108,921	2004,424,242	0,00,255,409,380,087,456	9	Cutting edge fields	1
ECONOMIC GROWTH	93	1,647,943,282	2002,741,935	− 0,0,146,503,144,173,161	9	Mainstream fields	1
ENERGY STORAGE TECHNOLOGIES	4	0,063,811,283	2010	0,0,937,259,664,035,621	16	Emerging fields	1
ENVIRONMENTAL DEGRADATION	5	0,082,640,742	2012	Inf	16	Emerging fields	1
ENVIRONMENTAL POLICY	13	0,237,789,171	1999,769,231	− 0,0,342,945,968,589,968	16	Specification fields	1
ENVIRONMENTAL PROTECTION	18	0,280,151,617	1997,555,556	− 0,0,348,336,446,107,098	16	Mainstream fields	1
ENVIRONMENTAL R&D	42	0,602,500,997	1998,333,333	− 0,0,360,011,102,058,357	10	Mainstream fields	1
ENVIRONMENTAL SCIENTIFIC AFFAIRS	4	0,127,301,587	1994,25	− 0,037,037,037,037,037	15	Specification fields	
ENVIRONMENTAL TECHNOLOGIES	37	0,600,442,058	2000,27,027	− 0,0,313,055,215,223,788	16	Mainstream fields	1
FINANCIAL CRISES	15	0,269,204,212	2009,8	Inf	7	Emerging fields	1
FINANCIAL INSTITUTIONS	19	0,268,992,611	1997,736,842	− 0,0,321,019,172,013,391	7	Mainstream fields	1
FINANCIAL MARKETS	21	0,486,720,094	2004,619,048	− 0,0,151,452,874,901,872	7	Mainstream fields	1
FINANCIAL SERVICES	10	0,177,380,952	1994,7	− 0,037,037,037,037,037	17	Specification fields	1
FINANCING INNOVATION	10	0,396,227,364	1996,7	− 0,0,356,719,757,799,131	15	Specification fields	1
FUEL CELL	16	0,283,006,881	2001,8125	− 0,037,037,037,037,037	16	Mainstream fields	
GENERAL POLICY	3	0,062,271,062	2007,333,333	− 0,0,141,093,474,426,808	1	Specification fields	
GLOBAL MARKETS	6	0,100,512,655	2006,166,667	0,0,297,000,532,036,093	13	Emerging fields	1
GLOBAL NETWORKS	4	0,061,022,121	2016	Inf	8	Emerging fields	1
GLOBAL VALUE CHAINS	8	0,16,397,593	2011,125	0,145,158,440,760,033	13	Emerging fields	1
GOVERNMENT FORESIGHT EXERCISES	7	0,119,365,079	1994,285,714	− 0,037,037,037,037,037	15	Specification fields	1
GOVERNMENT SERVICES	4	0,07,381,004	1998,5	− 0,037,037,037,037,037	7	Specification fields	1
GREEN GROWTH	57	1,200,346,327	2012,105,263	Inf	11	Cutting edge fields	1
HEALTH CARE SERVICES	5	0,081,781,377	2004,2	− 0,0,294,890,600,171,128	17	Specification fields	1
HEALTH SECTOR	3	0,05	2006	0	16	Emerging fields	1
HIGH TECH INDUSTRY	3	0,044,444,444	1999,333,333	− 0,0,231,481,481,481,481	14	Specification fields	1
HIGH TECHNOLOGIES	15	0,264,975,097	2006,866,667	0,00,895,303,161,108,388	13	Emerging fields	1
HIGHER EDUCATION	66	1,083,417,018	2008,136,364	0,0,451,923,503,115,839	10	Cutting edge fields	1
HUMAN RESOURCES DEVELOPMENT	8	0,145,028,217	2006,5	0,00,460,051,246,214,773	9	Emerging fields	1
IMPACT ASSESSMENT	96	1,862,531,385	2011,489,583	4,10,192,159,682,251	4	Cutting edge fields	
INDUSTRIAL DESIGN	3	0,051,388,889	1999,666,667	-0,025,025,025,025,025	17	Specification fields	1
INDUSTRY PARTNER	2	0,038,302,277	2015,5	Inf	9	Emerging fields	
INDUSTRY-SCIENCE LINKAGES	17	0,321,217,924	2006	0,000,921,473,542,198,539	0	Cutting edge fields	1
INFORMATION SOCIETY	9	0,166,247,227	2002,555,556	− 0,0,256,533,993,541,649	17	Specification fields	1
INFORMATION TECHNOLOGY	40	0,735,545,148	1998,8	− 0,0,309,595,550,521,648	17	Mainstream fields	1
INNOVATION-DRIVEN GROWTH	10	0,23,556,231	2012,1	Inf	2	Emerging fields	1
INNOVATION MANAGEMENT	11	0,24,583,602	2000,545,455	− 0,0,292,840,746,124,742	12	Specification fields	1
INNOVATION PERFORMANCE	73	1,333,558,866	2005,753,425	− 0,0,113,602,589,039,838	0	Mainstream fields	1
INNOVATION POLICY	460	8,588,809,823	2006,973,913	0,000,656,565,030,934,286	0	Cutting edge fields	1
INNOVATION PROCESSES	79	1,478,622,557	2002,025,316	− 0,0,275,382,727,209,115	12	Mainstream fields	1
INNOVATION STRATEGY	53	1,002,238,639	2008,188,679	0,253,671,026,967,128	1	Cutting edge fields	1
INNOVATION SYSTEM	140	2,521,269,312	2004,921,429	− 0,0,084,086,873,235,497	0	Mainstream fields	1
INTELLECTUAL ASSETS	52	0,883,067,687	2005,211,538	− 0,016,938,842,887,482	2	Mainstream fields	1
INTELLIGENT MANUFACTURING SYSTEMS	15	0,320,924,494	1996,266,667	− 0,037,037,037,037,037	15	Specification fields	1
INTEREST RATE	2	0,027,973,396	1997	− 0,037,037,037,037,037	7	Specification fields	
INTERNATIONAL COOPERATION	182	3,592,497,163	1997,725,275	− 0,0,321,791,964,999,185	3	Mainstream fields	1
INTERNATIONAL PUBLIC–PRIVATE PARTNERSHIPS	4	0,100,641,026	2002,25	− 0,037,037,037,037,037	15	Specification fields	1
INTERNATIONAL R&D COLLABORATION	14	0,238,525,451	2006,5	0,34,997,117,206,161	8	Emerging fields	1
INTERNATIONAL STUDENT	4	0,070,175,439	2007	Inf	10	Emerging fields	
INTERNATIONAL TECHNOLOGICAL CO-OPERATION	2	0,029,030,911	1999	− 0,037,037,037,037,037	3	Specification fields	1
INTERNATIONAL TRADE	13	0,285,724,212	1998,769,231	− 0,0,324,268,830,006,395	13	Specification fields	1
INTELLECTUAL PROPERTY RIGHTS	283	5,421,313,641	2003,056,537	− 0,0,238,540,107,085,683	8	Mainstream fields	1
JOB CREATION	102	1,771,333,461	1995,95,098	− 0,0,361,480,571,015,458	9	Mainstream fields	1
KNOW-HOW	21	0,381,381,571	2001,904,762	− 0,0,188,873,983,397,118	12	Mainstream fields	1
KNOWLDGE TRANSFER	17	0,330,987,547	2010,529,412	0,698,230,201,764,858	3	Cutting edge fields	
KNOWLEDGE-BASED ECONOMY	51	0,956,181,624	1998,862,745	-0,0,346,165,818,833,745	8	Mainstream fields	1
KNOWLEDGE CREATION	20	0,400,110,839	2001,9	− 0,0,268,141,256,830,698	12	Mainstream fields	1
KNOWLEDGE ECONOMY	14	0,271,547,477	2010,571,429	0,171,611,738,195,809	8	Emerging fields	1
KNOWLEDGE NETWORKS AND MARKETS	9	0,170,466,124	2010,777,778	Inf	15	Emerging fields	1
KNOWLEDGE TRANSFER	159	2,80,623,912	2011,937,107	0,0,423,283,194,688,183	3	Cutting edge fields	1
KNOWLEDGE TRIANGLE	135	2,620,373,757	2015,303,704	Inf	2	Cutting edge fields	1
LINEAR MODEL	9	0,146,593,665	1999,555,556	− 0,0,322,860,310,054,433	12	Specification fields	
LOCAL GOVERNMENT	5	0,089,403,471	2014,4	Inf	14	Emerging fields	
MARKET FAILURES	40	0,673,043,799	1998,7	-0,0,340,259,490,876,827	14	Mainstream fields	1
MEDICAL DEVICE	1	0,016,666,667	2006	0	17	Emerging fields	
NATIONAL STI GOVERNANCE	13	0,247,540,984	2009,692,308	Inf	0	Emerging fields	1
NATIONAL SYSTEM OF INNOVATION	238	4,518,076,291	1999,252,101	− 0,029,761,190,136,098	5	Mainstream fields	1
NEW PUBLIC MANAGEMENT	7	0,108,884,074	2007,142,857	0,0,307,772,561,293,687	4	Emerging fields	1
OPEN DATA	3	0,063,829,787	2013	Inf	2	Emerging fields	1
OPEN INNOVATION	163	2,855,282,933	2008,01,227	0,0,967,697,257,858,878	2	Cutting edge fields	1
OPEN SCIENCE	72	1,454,401,669	2014,083,333	Inf	2	Cutting edge fields	1
PHARMACEUTICAL BIOTECHNOLOGY	8	0,117,105,263	2003,75	− 0,037,037,037,037,037	15	Specification fields	1
POLICY INSTRUMENT	45	0,771,418,601	2009,888,889	0,0,356,667,393,202,722	0	Cutting edge fields	
POLICY MIX	129	2,379,159,162	2009,170,543	0,0,198,561,127,489,444	0	Cutting edge fields	1
PRIORITY SETTING	51	0,79,149,544	2008,137,255	0,226,706,487,181,874	4	Cutting edge fields	
PRIVATE INVESTMENT	11	0,189,049,308	2003,636,364	− 0,015,124,434,167,915	6	Specification fields	1
PRODUCTION ENGINEERING	1	0,015,873,016	1994	− 0,037,037,037,037,037	17	Specification fields	
PUBLIC AND PRIVATE RESOURCE	8	0,135,856,852	2009,5	0,0,510,789,049,919,485	5	Emerging fields	
PUBLIC AND PRIVATE SECTOR	28	0,510,095,026	2005,035,714	− 0,012,510,620,901,744	3	Mainstream fields	
PUBLIC FUNDING	27	0,539,277,699	2004,37,037	− 0,0,159,616,958,067,419	10	Mainstream fields	1
PUBLIC INTEREST	13	0,211,587,951	2014,461,538	0,521,506,824,001,479	8	Emerging fields	1
PUBLIC POLICY	45	0,822,212,426	2002,977,778	− 0,0,181,309,653,243,488	5	Mainstream fields	1
PUBLIC PRIVATE PARTNERSHIP	190	3,601,083,935	2003,442,105	− 0,0,273,108,271,709,256	5	Mainstream fields	1
PUBLIC PROCUREMENT	23	0,401,549,794	2007,956,522	0,0,246,218,668,335,072	3	Cutting edge fields	1
PUBLIC RESEARCH	193	3,491,233,808	2007,124,352	0,0,115,962,287,170,937	10	Cutting edge fields	1
PUBLIC SECTOR	77	1,324,999,371	2008,051,948	0,0,138,621,298,803,768	3	Cutting edge fields	1
PUBLIC SECTOR BODY	3	0,046,473,171	2002,333,333	− 0,037,037,037,037,037	12	Specification fields	
PUBLIC SUPPORT	77	1,379,318,427	2005,805,195	− 0,00,497,447,789,333,855	10	Mainstream fields	1
R&D ACTIVITIES	45	0,73,380,356	2004,355,556	0,00,151,061,327,809,132	14	Cutting edge fields	1
R&D EXPENDITURE	55	0,884,901,592	2003,145,455	-0,0,119,573,087,775,902	6	Mainstream fields	1
R&D INTENSITY	67	1,035,761,288	2011,865,672	0,053,554,151,093,938	6	Cutting edge fields	1
R&D INVESTMENT	49	0,844,597,218	2011,22,449	0,110,458,287,920,879	6	Cutting edge fields	1
R&D TAX POLICY	297	4,982,480,803	2004,676,768	0,0,103,310,116,888,676	6	Cutting edge fields	1
REGIONAL INNOVATION POLICY	7	0,168,610,094	2005,428,571	− 0,0,246,227,211,822,249	4	Specification fields	1
REGIONAL STRATEGY	7	0,166,666,667	2012	Inf	1	Emerging fields	1
REGULATORY REFORM	38	0,60,872,405	1998,157,895	− 0,0,337,423,296,618,728	3	Mainstream fields	1
RENEWABLE ENERGY	22	0,356,524,737	2006,818,182	0,0,308,627,873,021,742	16	Cutting edge fields	1
RESEARCH-BASED ENTREPRENEURSHIP	4	0,057,971,014	2019	Inf	11	Emerging fields	1
RESEARCH INSTITUTES	22	0,323,168,061	1999,863,636	-0,0,277,816,219,226,409	3	Mainstream fields	1
RESEARCH POLICY	27	0,470,920,801	2004,518,519	0,00,269,294,542,457,128	8	Cutting edge fields	1
RESEARCH SYSTEM	9	0,162,719,936	2007,888,889	0,0,510,408,863,779,455	10	Emerging fields	1
SCIENCE POLICY	15	0,287,292,338	2006,866,667	0,00,747,295,747,329,942	8	Emerging fields	1
SCIENTIFIC COMMUNITY	10	0,179,370,303	2005,6	− 0,0,158,829,639,265,361	3	Specification fields	1
SCIENTIFIC RESEARCH	27	0,500,542,807	2007,185,185	0,000,391,853,444,502,457	3	Cutting edge fields	1
SERVICE	512	8,848,855,913	1999,189,453	− 0,0,286,122,931,499,354	13	Mainstream fields	
SMALL ECONOMIES	7	0,110,172,378	2003,428,571	− 0,00,841,967,171,732,775	7 lePara>	Specification fields	1
SMALL FIRMS	77	1,256,359,706	2000,688,312	− 0,0,289,547,232,518,203	7	Mainstream fields	1
SMART CITIES	15	0,248,389,694	2016,333,333	Inf	11	Emerging fields	1
SMART REGULATION	14	0,288,243,427	2015,785,714	Inf	11	Emerging fields	1
SMART SPECIALISATION	140	3,142,390,841	2012,028,571	Inf	1	Cutting edge fields	1
SOCIAL AND GLOBAL CHALLENGE	5	0,079,747,642	2011,2	Inf	2	Emerging fields	
SOCIAL CAPITAL	8	0,121,318,822	2007	0,123,630,672,926,448	12	Emerging fields	1
SOCIO-ECONOMIC IMPACT	14	0,234,341,087	2008,142,857	Inf	4	Emerging fields	1
SOFT TECHNOLOGY	1	0,013,888,889	1996	-0,037,037,037,037,037	13	Specification fields	
SOFTWARE	51	0,882,930,954	2001,098,039	-0,0,251,321,505,119,347	17	Mainstream fields	
STI GOVERNANCE	39	0,749,971,138	2009,205,128	Inf	4	Cutting edge fields	1
STI POLICY	129	2,458,533,777	2007,449,612	0,0,128,824,994,902,014	4	Cutting edge fields	1
STOCK MARKET	11	0,154,226,529	1996,727,273	− 0,037,037,037,037,037	7	Specification fields	1
STRUCTURAL CHANGE	18	0,337,551,227	2006,388,889	0,0,182,789,504,185,696	13	Cutting edge fields	1
SUSTAINABLE CITIES	4	0,068,780,622	2016,25	Inf	11	Emerging fields	1
SUSTAINABLE DEVELOPMENT	99	1,767,598,456	2004,232,323	-0,0,255,062,186,332,828	11	Mainstream fields	1
SUSTAINABLE GROWTH	30	0,45,936,619	2015,733,333	0,249,183,614,839,871	9	Cutting edge fields	1
SYSTEMIC INNOVATION	141	2,794,856,585	2013,631,206	5,10,158,626,805,999	2	Cutting edge fields	1
SYSTEM TRANSFORMATION	38	0,74,281,991	2016,236,842	Inf	2	Cutting edge fields	1
SYSTEMIC INNOVATION POLICY	2	0,026,315,789	2004	− 0,037,037,037,037,037	15	Specification fields	1
TECHNOLOGICAL COMPETENCE	3	0,045,546,559	2004,333,333	− 0,037,037,037,037,037	14	Specification fields	1
TECHNOLOGICAL DEVELOPMENT	74	1,393,196	2001,081,081	− 0,0,261,141,140,107,565	3	Mainstream fields	1
TECHNOLOGICAL INNOVATION	31	0,540,019,445	1999,870,968	− 0,0,205,103,557,920,236	12	Mainstream fields	1
TECHNOLOGICAL STRATEGIES	5	0,083,492,063	1994,2	− 0,037,037,037,037,037	15	Specification fields	1
TECHNOLOGY MANAGEMENT	12	0,224,253,519	1997,666,667	− 0,0,338,936,300,930,044	15	Specification fields	1
SCIENCE, TECHNOLOGY AND INNOVATION POLICY	81	1,442,455,346	1997,62,963	− 0,0,360,055,389,507,165	5	Mainstream fields	1
TECHNOLOGY DIFFUSION	101	1,713,724,584	1996,930,693	− 0,0,363,187,549,716,071	12	Mainstream fields	1
TECHNOLOGY DIFFUSION PROGRAMME	63	1,090,864,632	1996,587,302	− 0,037,037,037,037,037	8	Mainstream fields	
TECHNOLOGY FORESIGHT	29	0,501,467,778	1999,517,241	− 0,0,297,647,902,336,597	4	Mainstream fields	1
TECHNOLOGY INVESTMENT	5	0,133,928,571	1995,6	− 0,037,037,037,037,037	7	Specification fields	1
TECHNOLOGY POLICY	178	3,564,811,283	2001,505,618	− 0,0,224,337,253,442,271	2	Mainstream fields	1
TECHNOLOGY POLICY OCEANS	4	0,127,301,587	1994,25	− 0,037,037,037,037,037	15	Specification fields	
TECHNOLOGY TRANSFER	66	1,199,381,852	2003,484,848	− 0,0,166,017,607,126,497	3	Mainstream fields	1
TECHNOLOGY TRANSFER OFFICE	11	0,197,852,727	2011,909,091	0,0,661,928,076,142,029	3	Emerging fields	
TRANSITION MANAGEMENT	8	0,163,152,759	2009,875	0,167,633,716,957,847	2	Emerging fields	1
USER-DRIVEN INNOVATION	5	0,083,382,753	2007	0,148,257,968,865,826	8	Emerging fields	1
VALUE CHAINS	19	0,347,554,156	2009,578,947	0,0,638,985,161,893,207	13	Cutting edge fields	1
VALUE CREATION	14	0,224,358,974	2005,142,857	− 0,0,189,716,633,436,046	15	Specification fields	1
VENTURE CAPITAL	100	1,597,645,571	1999,93	− 0,0,250,311,461,667,695	7	Mainstream fields	1
VENTURE CAPITAL FUND	16	0,239,254,386	1998,3125	− 0,0,276,484,862,951,028	7	Mainstream fields	

## Footnotes

1 For details, see: http://www.oecd.org/sti/inno/working-group-on-innovation-and-technology-policy.htm#:~:text=The%20OECD%20Working%20Party%20on,contributions%20to%20innovation%20and%20economic. Accessed 13 February 2021.

2 The authors wish to thank Dominque Guellec, Observatoire des Sciences et Techniques and Caroline Paunov, Organization for Economic Cooperation and Development, for their advice on the article.

3 The authors wish to thank Pavel Bakhtin for his advice on methodology.

4 The system is based on in-depth natural language processing (NLP). iFORA draws on an integrated database of diverse information and data sources. Its database hosts more than 500 million documents, including 120 million scientific publications in all research areas from Microsoft Academic Graph and CrossRef databases, 100 million WIPO, USPTO and Russian Patent Office patents, STI and market-related 40 million international and 10 million Russian news reports, as well as grant applications and grants supported by national and international science fondations, analytical and strategic documents and other sources of information. Data collection from a variety of open sources is carried out by parallel (multi-threaded and distributed) launch of software data extraction processes. The results generated by the iFORA system are used to produce a comprehensive and objective web of interconnected trends, drivers, barriers, and technologies to give an overview of the whole landscape of the sphere of interest in one dashboard. It maps the diversified dynamics of issues over decades when some of them still remain in the mainstream even though being affected by regular adjustments and refinements while others disappear under the pressures of newly emerged challenges and allied solutions. Success cases include semantic S&T landscape maps highlighting growing and fading topics, maps of national S&T competences and centers of excellence, and gap analyses for regulations and legislation. Valuable iFORA products are also collections of weak signals and’wild cards’ with potentially disruptive effects, and forward-looking timelines containing provisional future events as consequences of decisions previously made. For further details, see: https://issek.hse.ru/data/2020/05/28/1550143786/iFORA_brochure.pdf

## References

[CR1] Alfranseder, E., & Dzhamalova, V. (2014). The impact of the financial crisis on innovation and growth: Evidence from technology research and development. Knut Wicksell Working Paper 2014:8, The Knut Wicksell Centre for Financial Studies, Lund University, School of Economics and Management. https://www.nek.lu.se/media/kwc/working-papers/2014/kwc-wp-2014-8.pdf, Retrieved 20.05. 2022

[CR2] Arora S, Li Y, Liang Y, Ma T, Risteski A (2016). A latent variable model approach to pmi-based word embeddings. Transactions of the Association for Computational Linguistics.

[CR3] Bakhtin P, Saritas O, Chulok A, Kuzminov I, Timofeev A (2017). Trend monitoring for linking science and strategy. Scientometrics.

[CR4] Bakhtin P, Khabirova E, Kuzminov I, Thurner T (2020). The future of food production—a text-mining approach. Technology Analysis and Strategic Management.

[CR5] Carley SF, Newman NC, Porter AL, Garner JG (2018). An indicator of technical emergence. Scientometrics.

[CR6] Chen C (2006). CiteSpace II: Detecting and visualizing emerging trends and transient patterns in scientific literature. Journal of the Association for Information Science and Technology.

[CR7] Daim TU, Rueda G, Martin H, Gerdsri P (2006). Forecasting emerging technologies: Use of bibliometrics and patent analysis. Technological Forecasting and Social Change.

[CR8] De-Miguel-Molina B, Cunningham SW, Palop F (2017). Analyzing funding patterns and their evolution in two medical research topics. International Journal of Innovation and Technology Management.

[CR9] De Silva M, Gokhberg L, Meissner D, Russo M (2021). Addressing societal challenges through the simultaneous generation of social and business values: A conceptual framework for science-based co-creation. Technovation.

[CR10] Fagerberg J, Srholec M, Verspagen B, Hall BH, Rosenberg N (2012). Innovation and economic development. Handbook of the economics of innovation, 833–872, 1 edn.

[CR11] Fagerberg J, Morten F, Koson S (2012). Innovation: Exploring the knowledge base. Research Policy.

[CR12] Fagerberg J, Verspagen B (2009). Innovation studies—The emerging structure of a new scientific field. Research Policy.

[CR13] Gokhberg L (2020). Use AI to mine literature for policymaking. Nature.

[CR14] Gokhberg L, Kuzminov I, Khabirova E, Thurner T (2020). Advanced text-mining for trend analysis of Russia’s extractive industries. Futures.

[CR15] Gokhberg L, Meissner D, Gokhberg L, Meissner D, Sokolov A (2016). Seizing Opportunities for National STI Development. Deploying foresight for policy and strategy makers—creating opportunities through public policies and corporate strategies in science, technology and innovation.

[CR16] Hildebrandt C (2020). Ontology building for cyber–physical systems: Application in the manu-facturing domain. IEEE Transactions on Automation Science and Engineering.

[CR17] Huang Y, Zhang Y, Youtie J, Porter AL, Wang X (2016). How does national scientific funding support emerging interdisciplinary research: A comparison study of big data research in the US and China. PLoS ONE.

[CR18] Kincso I, Radosevic S (2017). EU research and innovation policies as factors of convergence or divergence after the crisis. Science and Public Policy.

[CR19] Kotis K, Vouros GA (2006). Human-centered ontology engineering: The HCOME methodology. Knowledge and Information Systems.

[CR20] Kuzhabekova A, Lee J (2018). International faculty contribution to local research capacity building: A view from publication data. Higher Education Policy.

[CR21] Li Y, Arora S, Youtie J, Shapira P (2018). Using web mining to explore Triple Helix influences on growth in small and mid-size firms. Technovation.

[CR22] Martin BR (1995). Foresight in science and technology. Technology Analysis & Strategic Management.

[CR23] Martin BR (2012). The evolution of science policy and innovation studies. Research Policy.

[CR24] McInnes, L., Healy, J., & Melville, J. (2018). Umap: Uniform manifold approximation and projection for dimension reduction. http://arxiv.org/abs/1802.03426.

[CR25] Melamud O, Goldberger J (2017). Information-Theory Interpretation of the Skip-Gram Negative-Sampling Objective Function. ACL.

[CR26] Mikolov, T., Chen, K., Corrado, G., & Dean, J. (2013a). Efficient estimation of word representations in vector space. http://arxiv.org/abs/1301.3781.

[CR27] Mikolov, T., Sutskever, I., Chen, K., Corrado, G. S., & Dean, J. (2013b). Distributed representations of words and phrases and their compositionality. *Advances in Neural Information Processing Systems*, 26.

[CR28] Morin, F., Bengio, Y. (2005) Hierarchical probabilistic neural network language model. In *Proceedings of the International Workshop on Artificial Intelligence and Statistics*, pp. 246–252.

[CR29] Morlacchi P, Martin BR (2009). Emerging challenges for science, technology and innovation policy research: A reflexive overview. Research Policy.

[CR30] Murtagh F, Contreras P (2012). Algorithms for hierarchical clustering: An overview. Wiley Interdisciplinary Reviews: Data Mining and Knowledge Discovery.

[CR31] OECD (2009). R&D in the economic crisis. In OECD Science, Technology and Industry Scoreboard 2009. *OECD Publishing*, Paris. 10.1787/sti_scoreboard-2009-5-en, Retrieved 18.05. 2022.

[CR32] Peia O, Romelli D (2022). Did financial frictions stifle R&D investment in Europe during the great recession?. Journal of International Money and Finance.

[CR33] Pereira C, Sousa C, Soares AL (2013). Supporting conceptualisation processes in collaborative networks: A case study on an R&D project. International Journal of Computer Integrated Manufacturing.

[CR34] Rehm, J. (2018). Ten years after the economic crash, R&D funding is better than ever. *Nature* 13 September 2018. https://www.nature.com/articles/d41586-018-06634-4, Retrieved 20.05. 2022

[CR35] Rotolo D, Hicks D, Martin BR (2015). What is an emerging technology?. Research Policy.

[CR36] Shapira P, Kwon S, Youtie J (2017). Tracking the emergence of synthetic biology. Scientometrics.

[CR37] Shen YC, Chang SH, Lin GT, Yu HC (2010). A hybrid selection model for emerging technology. Technological Forecasting and Social Change.

[CR38] Stapleton L (2006). Modes of reasoning in theories of the social impact of advanced technology: A critique of ERP systems in healthcare. Annual Reviews in Control.

[CR39] Verspagen B (2007). Mapping technological trajectories as patent citation networks: A study on the history of fuel cell research. Advances in Complex Systems.

[CR40] Ward JH (1963). Hierarchical grouping to optimize an objective function. Journal of the American Statistical Association.

[CR41] Youtie J, Bozeman B, Jabbehdari S, Kao A (2017). Credibility and use of scientific and technical information in policy making: An analysis of the information bases of the National Research Council’s committee reports. Research Policy.

[CR42] Zhang Y, Zhang G, Zhu D, Lu J (2017). Scientific evolutionary pathways: Identifying and visualizing relationships for scientific topics. Journal of the Association for Information Science and Technology.

[CR43] Zhang Y, Zhou X, Porter AL, Gomila JMV, Yan A (2014). Triple Helix innovation in China’s dye-sensitized solar cell industry: Hybrid methods with semantic TRIZ and technology roadmapping. Scientometrics.

